# Supplementary material for: Energy correlations of photon pairs generated by a silicon microring resonator probed by Stimulated Four Wave Mixing
Source: Sci Rep. 2016 Apr 1;6:23564. doi: 10.1038/srep23564 (PMC4817032; doi:10.1038/srep23564)
Supplement: Supplementary Information [file srep23564-s1.pdf]

Supplementary Information: Energy correlations of photon pairs generated by a silicon microring resonator probed by Stimulated Four Wave Mixing.

Davide Grassani[1], Angelica Simbula[1], Stefano Pirotta[1], Matteo Galli[1], Matteo Menotti[1], Nicholas C. Harris[2], Tom Baehr-Jones[3], Michael Hochberg[3], Christophe Galland[4], Marco Liscidini[1], and Daniele Bajoni[5,*]

[1]Dipartimento di Fisica, Università di Pavia, via Bassi 6, 27100 Pavia, Italy
[2]Department of Electrical Engineering and Computer Science, Massachusetts Institute of Technology, 77 Massachusetts Avenue, Cambridge, MA 02139, USA
[3]Coriant Advanced Technology Group, 1415 West Diehl Road, Naperville, IL 60563, United States
[4]Ecole Polytechnique Fédérale de Lausanne, SB-LPQM, CH-1015 Lausanne, Switzerland
[5]Dipartimento di Ingegneria Industriale e dell'Informazione, Università di Pavia, via Ferrata 1, 27100 Pavia, Italy
[*]daniele.bajoni@unipv.it

January 11, 2016

Active stabilization of the Fabry-Pérot filter

Exploiting the stimulated process to reconstruct the JSD, the resolution on the signal axis is given by the quasi-CW seed, which is about 2 pm in our case, while the resolution on the idler axis is given by a home-made Fabry-Pérot (FP) filter. The FP consists in two parallel UV fused silica broadband beamsplitters (BSs) placed inside two mirror mounts and aligned to be parallel by means of micrometer screws and the help of an IR camera. Each mirror mount is screwed on a three axis translator controlled in one direction by piezoelectric actuators to modify the spacing distance between the BSs (see Supplementary Fig. S1). The output beam exiting the sample is collected by a single mode fiber (SMF) and collimated by an objective at normal incidence on the FP. The output of the filter is then focused on a SMF by a second identical objective. The working range of the BSs is 1.2-1.6 μm and their reflectance is about 90 % at 45° of angle of incidence.

We set the spacing between the reflecting sides of the BSs, i.e. the FP length, at about 5 mm, leading to a free spectral range (FSR) of $\Delta\lambda \simeq 240$ pm as shown in Supplementary Fig. S2. The measured FWHM of the FP is $\delta\lambda \simeq 5$ pm, which is

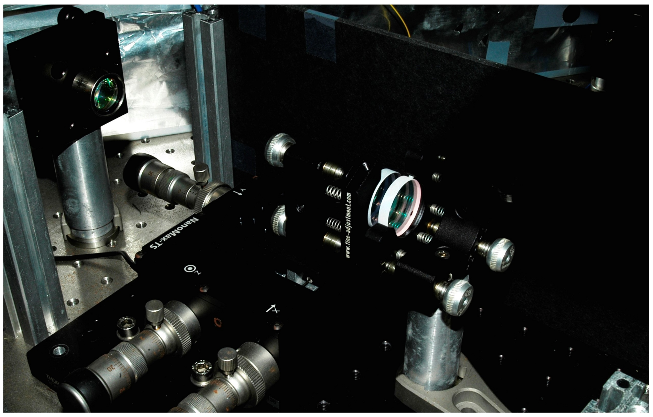

Supplementary Figure 1. Image of the Fabry-Pérot set-up.

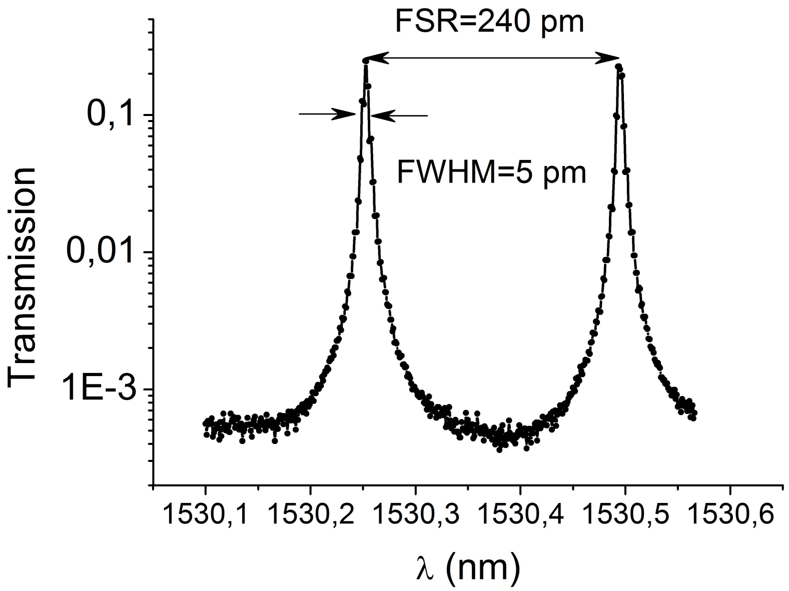

Supplementary Figure 2. Measured transmission spectrum of the Fabry-Pérot filter.

sufficient to sample in about 28 points the 140 pm wavelength range around the idler resonance. It is important to notice that the FSR has to be larger than the wavelength range spanned, or at least larger than the resonance width. The Finesse is thus $F = \Delta\lambda/\delta\lambda \simeq 50$, higher than the one expected from the reflectance of the BSs, probably because of the different behavior of the reflective coating at normal incidence. Considering the measured Finesse, the filter rejection is about 30dB, as confirmed in Supplementary Fig. S2.

In order to stabilize the FP length against environmental noise and change the filter wavelength, we implemented a remotely controlled active feedback loop on the piezoelectric actuators, using a reference tunable quasi-CW O-band laser. The reference laser is sent to the FP together with the C-band generated idler beam by a fiber wavelength division multiplexer (WDM) placed at the sample output and it is filtered out at the output of the FP (Fig. 1 in the main text). The working principle of the feedback is the following: we send a very small voltage modulation (dither) to the piezoelectric actuator which continuously modulates the FP length. The optical output of the FP filter is thus also modulated, but the modulation amplitude depends on the position of the control laser wavelength with respect to the FP resonance. In practice, the amplitude of the optical modulation (OAM) is the first order derivative of the FP transmission (or reflection) function, and it is linear across the resonance peak (or dip), where it is zero (see Supplementary Fig. S3). Then, considering laser wavelengths inside the FP resonance (gray region in Supplementary Fig. S3), farther is the reference laser wavelength from the FP peak (or dip), larger would be the OAM. We thus use the OAM as the input error function for a computer based P.I.D. algorithm, which sends a dc voltage feedback to the piezo actuator to compensate for length or refractive index fluctuations and stabilize the FP resonance on the reference laser line. The dither amplitude is very small and does not perturb the FB output, so that we can stabilize the FP resonance wavelength with about a pm of precision. Changing the reference laser wavelength would change the stabilization point and thus the filter wavelength, allowing filter tunability.

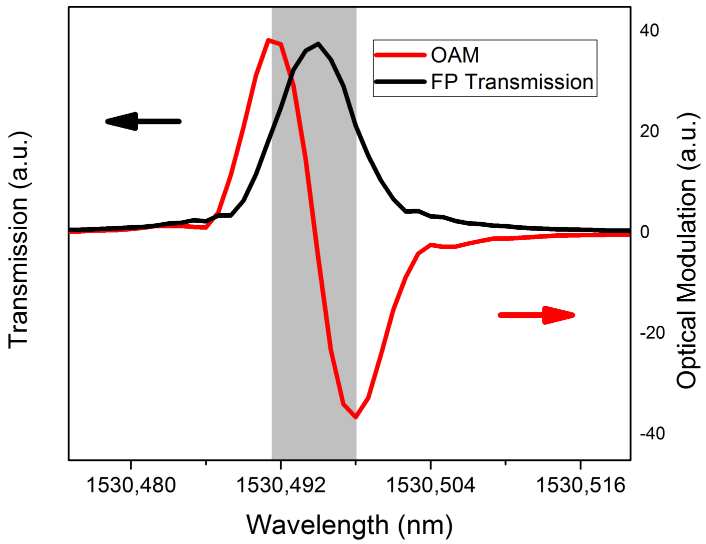

Supplementary Figure 3. Laser scan of one of the transmission resonances of the Fabry-Pérot (black line) superimposed to the amplitude of the optical modulation (OAM) given by the dither (red line). The gray region highlights the linear part of the OAM, used as error function for the P.I.D. feedback.

Experimental error on the Schmidt Number

The evaluation of K_{bound} has been performed as in [G. Harder, et al. Opt. Express 21, 13975 (2013)] starting from the experimental JSD. The uncertainty depends on two terms: (1) the signal-to-noise ratio; (2) the measurement resolution; The noise depends on several parameters, and it can be different in the case of pulse or in quasi-CW pumping regime. Yet, since here we are dealing with noise that is uncorrelated in frequency, the effect is that of reducing K_{bound}, for a K which is proportional to the correlations. On the contrary, the uncertainty associated with the measurement resolution can be estimated with a simple argument.

Effects of finite measurement resolution

In the case of SFWM in a ring resonator, one can assume the biphoton wavefunction can be written as the product of two gaussians of standard deviation $\sigma_+$and σ_-, where $(\sigma_+)_\omega$ and $(\sigma_-)_\omega$ are the standard deviation along the direction $\omega_1 + \omega_2 = 2\omega_{p0}$ and $\omega_1 - \omega_2 = \omega_{i0} - \omega_{s0}$, respectively [see J. Mower et al. PRA 87, 062322 (2013)]. Here ω_{p0}, ω_{s0}, and ω_{i0} are the center of the pump, signal, and idler resonance, respectively. In this case the Schmidt number is:

$$K \approx \frac{(\sigma_-)_\omega}{(\sigma_+)_\omega}. \qquad \text{(Supplementary Equation 1)}$$

In our experiment we work in wavelength rather than in frequency, but it is easy to show that

$$K \approx \frac{(\sigma_-)_\lambda}{(\sigma_+)_\lambda}, \qquad \text{(Supplementary Equation 2)}$$

where $(\sigma_+)_\lambda$ and $(\sigma_-)_\lambda$ are the standard deviation in wavelength.

The experimental error associated to the calculation of the Schmidt number comes from the uncertainties associate to our stimulated emission measurements, which are Δ_i and Δ_s on the idler and signal wavelength, respectively. The error on $(\sigma_+)_\lambda$ and $(\sigma_-)_\lambda$ can be calculated by standard propagation error theory as:

$$\Delta_\pm = \sqrt{(\Delta_i)^2 + (\Delta_s)^2}. \qquad \text{(Supplementary Equation 3)}$$

Similarly, since K is given by Supplementary Equation 2, we have that

$$\left(\frac{\Delta K}{K}\right)^2 = \left(\frac{\Delta_+}{(\sigma_+)_\lambda}\right)^2 + \left(\frac{\Delta_-}{(\sigma_-)_\lambda}\right)^2, \qquad \text{(Supplementary Equation 4)}$$

which gives

$$\Delta K = \frac{(\sigma_-)_\lambda}{(\sigma_+)_\lambda}\sqrt{\left(\frac{1}{(\sigma_-)_\lambda}\right)^2 + \left(\frac{1}{(\sigma_+)_\lambda}\right)^2}\sqrt{(\Delta_i)^2 + (\Delta_s)^2}. \qquad \text{(Supplementary Equation 5)}$$

Pulsed case

Our experimental setup has uncertainties $\Delta_i = 2$ pm and $\Delta_s = 4.5$ pm. In the case of a pump pulse that is spectrally wider than the pump resonance we have that $(\sigma_-)_\lambda \approx (\sigma_+)_\lambda$ [see L.G. Helt et al, OL 35, 3006 (2010)]. From our measurement we have $(\sigma_-)_\lambda \approx 50$ pm, which gives:

$$\Delta K \approx \frac{1}{(\sigma_-)_\lambda}\sqrt{(\Delta_i)^2 + (\Delta_s)^2} \approx \frac{1}{50}\sqrt{4 + 20.25} \approx 0.1. \qquad \text{(Supplementary Equation 6)}$$

Quasi-CW pump

In the case of a quasi-CW pump, we observe that the value of $(\sigma_-)_\lambda$ is limited by the setup resolution, in particular the expected $(\sigma_-)_\lambda \ll \sqrt{(\Delta_i)^2 + (\Delta_s)^2}$. Thus, $\sqrt{(\Delta_i)^2 + (\Delta_s)^2}$ is an upper bound for the expected $(\sigma_-)_\lambda$. This gives

$$K_{bound} > \frac{(\sigma_-)_\lambda}{\sqrt{(\Delta_i)^2 + (\Delta_s)^2}} \approx 10, \qquad \text{(Supplementary Equation 7)}$$

which is what one would expect for an ideal measurement without noise. In our case, we find $K_{bound} = 3.9$. The value is lower than expected, but still significantly larger than 1, which indicates correlations. This difference is attributed to the noise in the measurements, which is uncorrelated and thus tends to reduce the Schmidt number.
